# Identification of an HIV-1 BG Intersubtype Recombinant Form (CRF73_BG), Partially Related to CRF14_BG, Which Is Circulating in Portugal and Spain

**DOI:** 10.1371/journal.pone.0148549

**Published:** 2016-02-22

**Authors:** Aurora Fernández-García, Elena Delgado, María Teresa Cuevas, Yolanda Vega, Vanessa Montero, Mónica Sánchez, Cristina Carrera, María José López-Álvarez, Celia Miralles, Sonia Pérez-Castro, Gustavo Cilla, Carmen Hinojosa, Lucía Pérez-Álvarez, Michael M. Thomson

**Affiliations:** 1 Centro Nacional de Microbiología, Instituto de Salud Carlos III, Majadahonda, Madrid, Spain; 2 CIBER de Epidemiología y Salud Pública (CIBERESP), Madrid, Spain; 3 Hospital Universitario Lucus Augusti, Lugo, Spain; 4 Complejo Hospitalario Universitario de Vigo, Vigo, Pontevedra, Spain; 5 Hospital Universitario Donostia, San Sebastián, Spain; 6 Hospital Clínico Universitario de Valladolid, Valladolid, Spain; University of Malaya, MALAYSIA

## Abstract

HIV-1 exhibits a characteristically high genetic diversity, with the M group, responsible for the pandemic, being classified into nine subtypes, 72 circulating recombinant forms (CRFs) and numerous unique recombinant forms (URFs). Here we characterize the near full-length genome sequence of an HIV-1 BG intersubtype recombinant virus (X3208) collected in Galicia (Northwest Spain) which exhibits a mosaic structure coincident with that of a previously characterized BG recombinant virus (9601_01), collected in Germany and epidemiologically linked to Portugal, and different from currently defined CRFs. Similar recombination patterns were found in partial genome sequences from three other BG recombinant viruses, one newly derived, from a virus collected in Spain, and two retrieved from databases, collected in France and Portugal, respectively. Breakpoint coincidence and clustering in phylogenetic trees of these epidemiologically-unlinked viruses allow to define a new HIV-1 CRF (CRF73_BG). CRF73_BG shares one breakpoint in the envelope with CRF14_BG, which circulates in Portugal and Spain, and groups with it in a subtype B envelope fragment, but the greatest part of its genome does not appear to derive from CRF14_BG, although both CRFs share as parental strain the subtype G variant circulating in the Iberian Peninsula. Phylogenetic clustering of partial *pol* and *env* segments from viruses collected in Portugal and Spain with X3208 and 9691_01 indicates that CRF73_BG is circulating in both countries, with proportions of around 2–3% Portuguese database HIV-1 isolates clustering with CRF73_BG. The fact that an HIV-1 recombinant virus characterized ten years ago as a URF has been shown to represent a CRF suggests that the number of HIV-1 CRFs may be much greater than currently known.

## Introduction

HIV-1 is characterized for its high genetic diversity, derived from elevated mutation and recombination rates. Through these mechanisms, HIV-1 group M, responsible for the pandemic, has evolved into multiple genetic forms, named subtypes, subsubtypes, and circulating and unique recombinant forms (CRFs and URFs). Among these, subtype B is the predominant HIV-1 clade circulating in Western European countries. However, nonsubtype B viruses are common in most of them, although these usually have been associated to travel and migration and have been acquired in or are epidemiologically linked to other continents, most frequently sub-Saharan Africa [1–3]. Notable exceptions to this rule are Portugal, where a subtype G variant circulates at a high prevalence among the local HIV-1-infected population [4,5], and the Spanish region of Galicia, north of Portugal, where the mentioned subtype G variant [6,7] and a recently originated subtype F cluster [8] are spreading locally. Recombination of the Spanish-Portuguese or Iberian subtype G (G_Ib_) variant with subtype B has given rise to CRF14_BG, initially identified among injecting drug users (IDUs) in Galicia [6,7], but also circulating in other Spanish regions [9,10] and in Portugal [5,11,12], and to diverse unique BG recombinant viruses [6,7,13,14].

One of the BG recombinant viruses different from CRF14_BG of presumable Iberian ancestry was collected in Germany in 2001 from an IDU who had resided in Portugal [15]. This virus, designated 9196_01, was characterized in the near full-length genome, with most of it deriving from subtype G, with three subtype B segments located in *pol* [∼230 nucleotides (nt)], *vif* (∼160 nt), and *env* (∼1.9 kb) genes. In phylogenetic trees, the 3’ half of the subtype B fragment of the envelope of 9196_01 clustered with CRF14_BG references. Subtype G segments of 9196_01 also clustered with CRF14_BG, although this might reflect a common parental G_Ib_ strain. Here we show that 9196_01 represents a new CRF by characterizing the near full-length genome sequence of a second and partial genome sequences of another three epidemiologically-unlinked viruses showing coincident mosaic structures and phylogenetic clustering with 9196_01.

## Materials and Methods

### Samples

For this study, we used plasma samples from HIV-1-infected individuals from different regions of Spain. Most samples were from Galicia and Basque Country, in Northwest and North Spain, respectively, from whose public hospitals, since 1999 in Galicia and 2001 in Basque Country, we regularly receive HIV-1 samples, including those from newly diagnosed infections, which are phylogenetically characterized. Samples from other Spanish regions (Navarra, Castilla y León, Madrid, Castilla-La Mancha, and Extremadura), were also analyzed with a more limited coverage.

The study was approved by the Bioethics and Animal Well-being Committee of Instituto de Salud Carlos III, Majadahonda, Madrid, Spain, report number CEI PI 51_2011-v2. Written informed consent was obtained from all participants in the study.

### RNA Extraction

RNA was extracted from 1 ml plasma using Nuclisens Easy MAG kit (bioMérieux, Marcy l’Etoile, France) following the manufacturer’s instructions.

### RT-PCR Amplification and Sequencing

The HIV-1 protease-reverse transcriptase (PR-RT) segment of *pol* (HXB2 positions 2253–3629) and the C2-V3-C3 segment of *env* (HXB2 positions 7013–7647) were amplified by RT-PCR, using One Step RT-PCR Kit (Qiagen, Hilden, Germany), followed by nested PCR, using Biotaq DNA polymerase (Bioline, London, UK), and sequenced, as described [16]. Near full-length genome (∼9 kb) amplification by RT-PCR followed by nested PCR in four overlapping fragments of 1.8–3 kb and sequencing was done as described [7,16,17] (the detailed protocol is available at EURIPRED’s web site: http://www.euripred.eu/information-products/sops.html).

Sequences were deposited in GenBank under accessions KM248760-KM248766, KM892492.

### Phylogenetic Sequence Analyses

Sequences were aligned with MAFFT v.7 [18]. Phylogenetic trees were constructed via maximum likelihood (ML) with RAxML v.7.2.7 [19], applying the general time reversible substitution model with gamma-distributed among-site rate heterogeneity and a proportion of invariant sites (GTR+G+I), with assessment of node support by bootstrapping. Phylogenetic trees were also constructed by Bayesian inference with MrBayes v3.2.5 [20], using the GTR+G+I substitution model. For each dataset, two simultaneous independent runs were performed, with eight chains, sampling every 500 generations. The analyses were run until both runs had reached convergence, as determined by an average standard deviation of split frequencies <0.01. Node support was derived from a majority-rule consensus of trees sampled from the posterior distribution, discarding the first 50% as burn-in.

Recombination was analyzed by bootscanning with Simplot v3.5 [21], using a 250 nt window and a 20 nt step, with tree construction by the neighbor-joining method applying the Kimura two-parameter substitution model. Precise breakpoint locations were analyzed with jpHMM [22].

Relationships of the sequenced viruses with sequences deposited at the Los Alamos HIV Sequence Database [23] were assessed through BLAST searches followed by phylogenetic analyses incorporating the 50 sequences with the highest similarity scores.

### tMRCA Estimation

To estimate the time of the most recent common ancestor (tMRCA) of the identified clade, we used a Bayesian Markov Chain Monte Carlo (MCMC) coalescent method as implemented in BEAST v1.8.1 [24]. For this analysis, we used all PR-RT sequences of the identified clade with known sample collection year together with PR-RT sequences from all available subtype G near full-length genome sequences from the Los Alamos HIV Sequence Database. We chose an HKY substitution model with gamma-distributed among-site rate heterogeneity and two partitions in codon positions (1st+2nd; 3rd) [25], an uncorrelated lognormal relaxed clock model and a Bayesian skyline plot demographic model [26]. Each MCMC chain was run for 100 million generations, sampling every 5,000. MCMC convergence and effective sample sizes (ESS) were checked with Tracer v.1.5 (http://tree.bio.ed.ac.uk/software/tracer/), ensuring that the ESS of each parameter was >200. Results were summarized with a maximum clade credibility (MCC) tree, using TreeAnnotator v1.5.3, after removal of a 50% burn-in. The MCC tree was visualized with FigTree v1.3.1. (http://tree.bio.ed.ac.uk/software/figtree/). Parameter uncertainty was summarized in the 95% highest posterior density (HPD) intervals.

## Results

### Viruses from Portugal and Spain Are Related to the HIV-1 BG Recombinant Virus 9196_01 in PR-RT and *env* V3 Region

**Fig 1:**
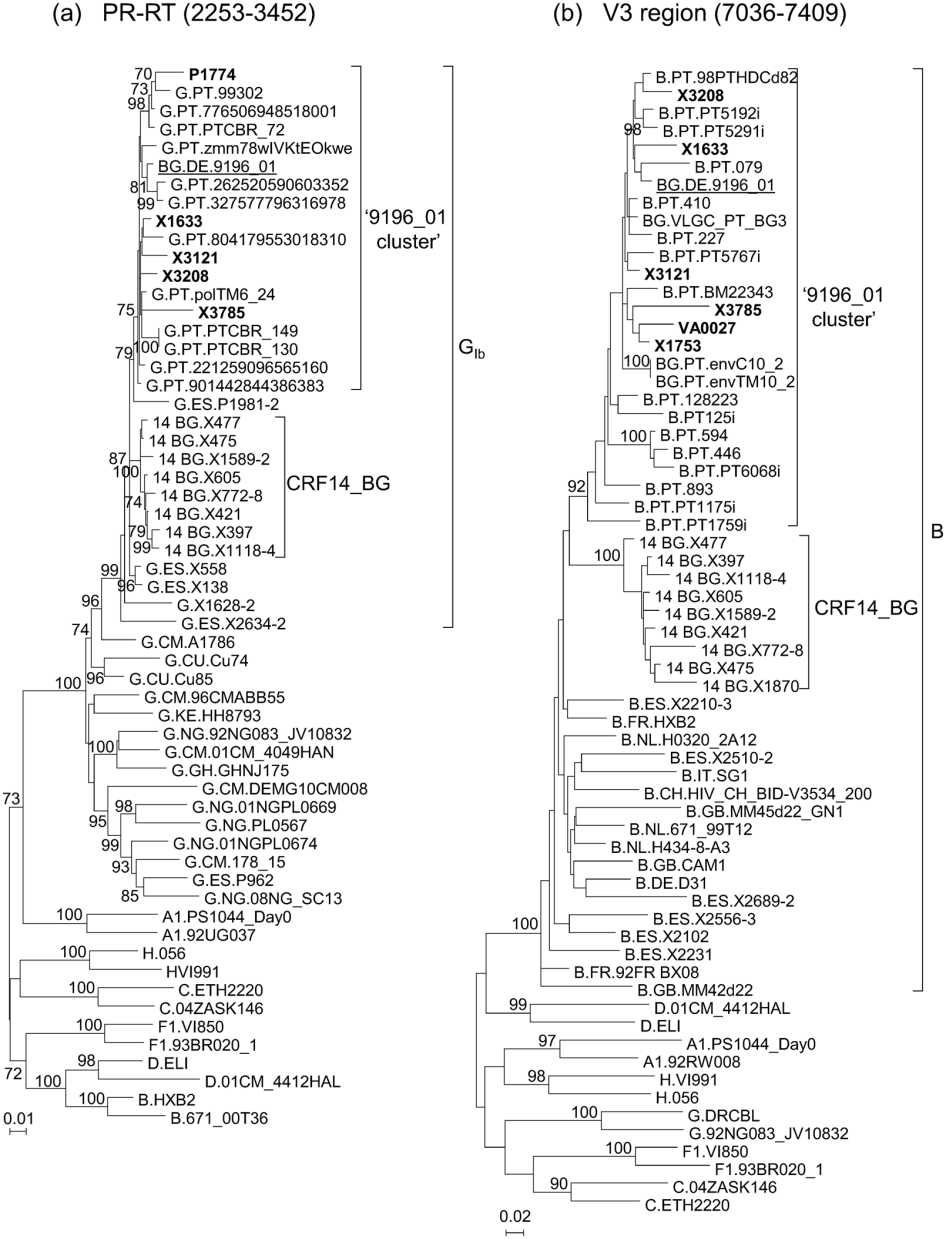
ML trees of sequences of HIV-1 isolates from databases (all from Portugal) or obtained by us (from Spain) clustering with the BG recombinant virus 9196_01 in (a) PR-RT and/or the (b) V3 region. Sequences obtained in this study are in bold type. HXB2 positions delimiting the analyzed segments are in parentheses. Countries of collection of database viruses of subtype G in PR-RT and of subtype B in V3 are indicated with the two-letter ISO code. Only bootstrap values ≥70% are shown.

In analyses of HIV-1 PR-RT and V3 sequences from samples collected in different regions of Spain, from a total of more than 8,000 individuals whose samples were studied, we identified a subtype G PR-RT cluster of five viruses which in the V3 region were of subtype B and formed a cluster with two other sequences of Spanish viruses obtained by us, branching apart from CRF14_BG viruses (Fig 1, S1 Fig). Through BLAST searches in databases using sequences of PR-RT and the V3 region and subsequent phylogenetic analyses, we found that they had high similarity scores with the 9196_01 BG recombinant strain and with several Portuguese viruses, clustering with them in both analyzed segments in ML (Fig 1) and Bayesian phylogenetic trees [with posterior probabilities (PP) of 0.96 and 1 for PR-RT and V3 segments, respectively] (S1 Fig). The total number of viruses clustering with 9196_01 in PR-RT and/or V3 region was 38, from different individuals, 31 of which were from Portugal (12 in PR-RT and 19 in the V3 region) and seven from Spain (five from Galicia) (four in PR-RT and the V3 region, one only in PR-RT, and two only in the V3 region). Epidemiological data of the individuals with viruses collected in Spain (S1 Table) indicate that two were Portuguese and four of six with reported transmission routes were IDUs. Data of viruses collected in Portugal, whose sequences are deposited in databases, available in GenBank entries or published papers [4,5,11], are also shown in S1 Table. Transmission category was available for 13, and it was IDU in 12.

### Analysis of the Near Full-Length Genome of a Virus from Spain (X3208) Reveals a Mosaic Structure Coincident with that of 9196_01

**Fig 2:**
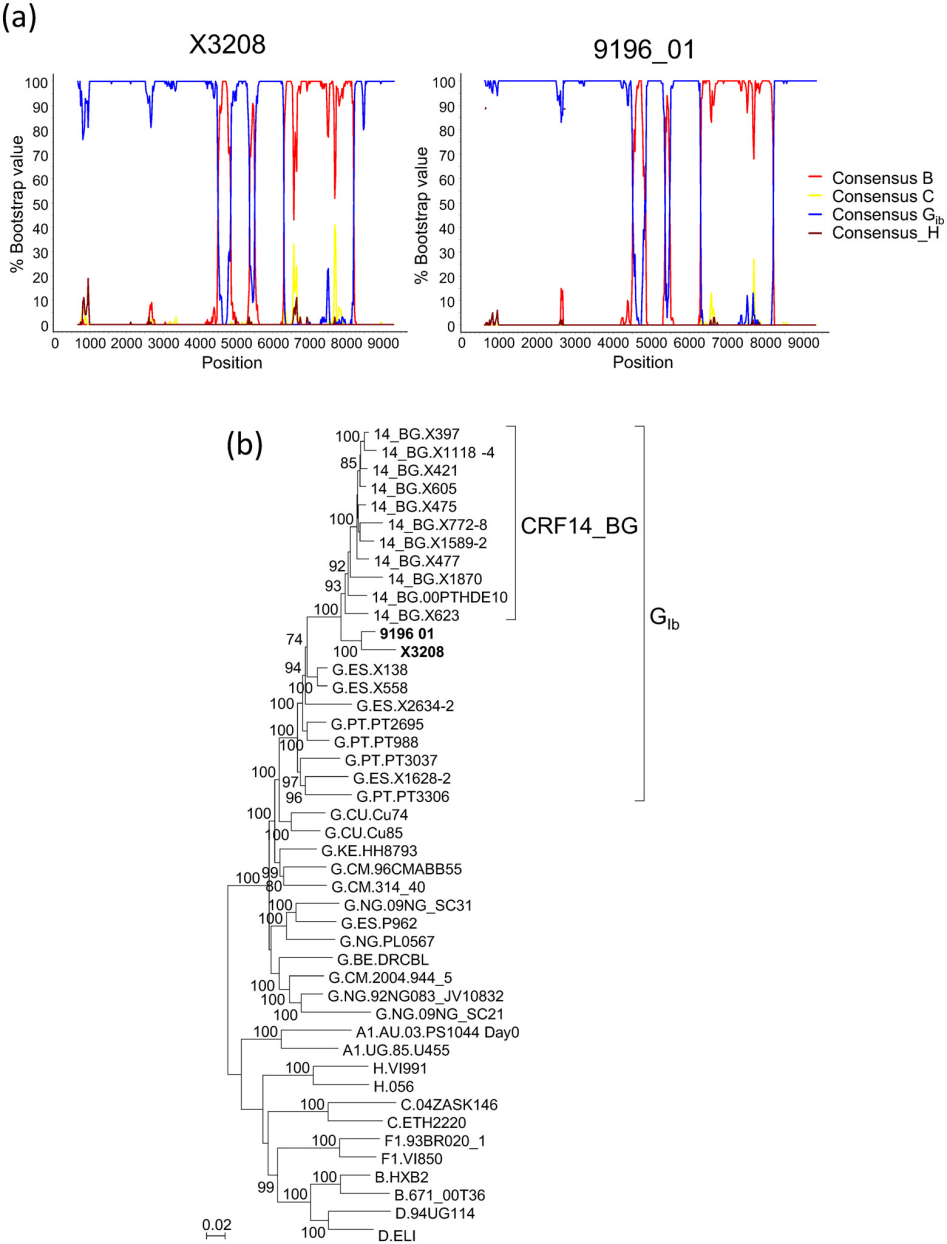
Analysis of the near full-length genome sequence of X3208. **(a) Bootscan analyses of X3208 and 9196_01.** Positions in the horizontal axis correspond to the midpoint of the sliding window in the HXB2 proviral genome sequence. (b) ML tree of near full-length genomes of X3208 and 9196_01, analyzed with subtype G viruses from Spain, Portugal, and other countries, and CRF14_BG references. Countries of collection of subtype G sequences are indicated with the two-letter ISO code. Only bootstrap values ≥70% are shown.

We obtained the near full-length genome sequence of one of the Galician viruses clustering with 9196_01 in PR-RT and V3 segments, X3208, collected in 2011 in the city of Lugo from a heterosexually-infected Spanish woman newly diagnosed of HIV-1 infection, in order to determine whether its mosaic structure coincided with that of 9196_01. In the bootscan analysis, X3208 was predominantly of subtype G, with three subtype B fragments, two short ones in the integrase coding sequence and in *vif*, respectively, and a larger one, comprising most of *env*, delimited by breakpoints mear the 5’ end of gp120 and in gp41, respectively, showing a mosaic structure coincident with that of 9196_01 (Fig 2a). Coincidence of breakpoint locations between both viruses was confirmed in analyses with jpHMM (Table 1). The breakpoints at gp41 of X3208 and 9196_01 coincided with that of CRF14_BG, but those at gp120 were located around 120 nt upstream of that of CRF14_BG (Table 1). In the phylogenetic trees, X3208 and 9196_01 formed a cluster supported by a 100% bootstrap value and a Bayesian PP of 1, which was sister to the CRF14_BG clade, which was supported by bootstrap value of 93% and a PP of 1, with all these BG recombinant viruses branching in the G_Ib_ clade (Fig 2b, S2 Fig).

**Table 1:** Intersubtype breakpoint locations in HIV-1 BG recombinant viruses analyzed in this study, including all available CRF14_BG viruses sequenced in near full-length genomes, as determined with jpHMM.

Sample ID	Genes and positions in HXB2 genome of breakpoint locations					
	Integrase	Integrase	Vif	Vif	gp120	gp41
9196_01	4580±20	4847±16	5341±25	5515±22	6215±25	8271±44
X3208	4557±14	4847±16	5341±24	5503±10	6228±38	8296±34
X3121	4562±27	4847±16	n.a.	n.a.	n.a.	n.a.
753_G_0_Rennes	4560±25	4847±16	n.a.	n.a.	n.a.	n.a.
VLGC					6251±26	8289±27
CRF14_BG						
X397					6340±19	8289±27
X421					6340±19	8289±27
X475					6340±19	8289±27
X477					6340±19	8289±30
X605					6340±19	8290±28
X623					6341±18	8279±17
X1118-4					6339±18	8279±17
X1589-2					6333±26	8290±28
X1870					6339±17	8328±41
00PTHDE10					6341±18	8289±27

n.a.: sequence not available in the corresponding segment.

### Three Epidemiologically-Unlinked Viruses Analyzed in Partial Genome Sequences Show BG Mosaic Structures Coincident with 9196_01 and X3208 and Cluster with Them in Phylogenetic Trees, Allowing to Define a New HIV-1 CRF

**Fig 3:**
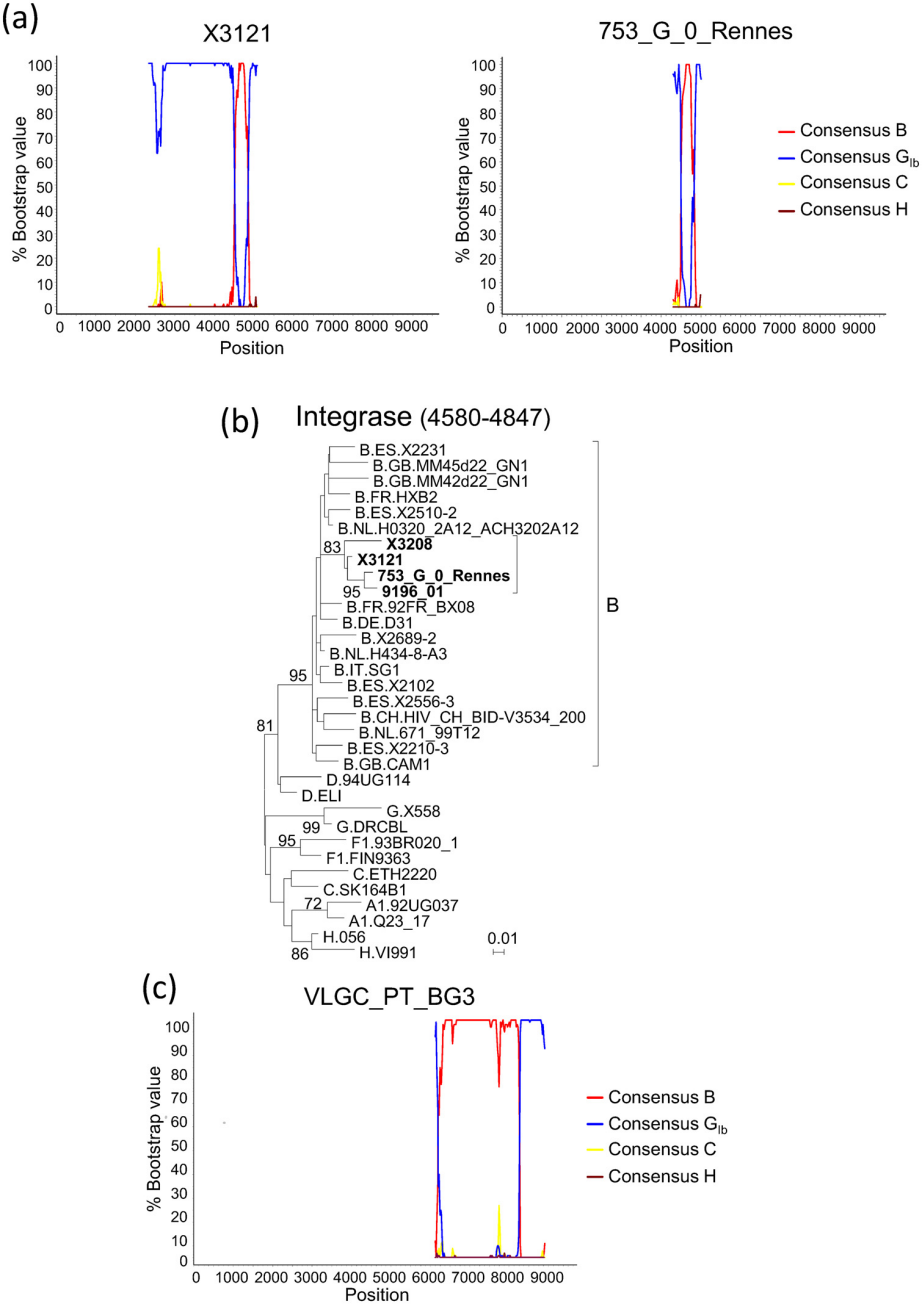
Analyses of partial genome sequences of BG recombinant viruses related to X3208 and 9196_01. (a) Bootscan analyses of *pol* fragments of X3121, from Spain, and 753_G_0_Rennes, from France. (b) ML tree of the integrase subtype B fragment of X3121 and 753_G_0_Rennes, showing clustering with 9196_01 and X3208. HXB2 positions delimiting the analyzed segment are in parentheses. Only bootstrap values ≥70% are shown. (c) Bootscan analysis of the envelope gene of VLGC_PT_BG3, from Portugal. In the bootscanning graphs, the position in the horizontal axis represents the midpoint of the sliding window in the proviral HXB2 genome.

In order to determine whether additional BG recombinant viruses had mosaic structures coincident with that of 9196_01 and X3208, we sequenced an ∼3 kb fragment in the 5’ half of the genome of another virus from Spain, X3121, which clustered with the mentioned recombinants in PR-RT and the V3 region (Fig 1, S1 Fig). X3121 was collected in 2011 in the Galician city of Vigo from a Spanish man who was an IDU and had no known epidemiological links with X3208. The sequenced fragment of X3121, comprising most of *pol* and the 5’ segment of *vif*, when analyzed by bootscanning, revealed the presence of a subtype B fragment in integrase (Fig 3a), delimited by breakpoints coincident with those of 9196_01 and X3208, a coincidence which was confirmed in the analysis with jpHMM (Table 1).

Through BLAST searches of the integrase subtype B fragments (HXB2 positions 4580–4847) of X3208 and 9196_01 in the HIV Sequence Database [24] and subsequent bootscan and phylogenetic analyses, we found one additional BG recombinant virus, named 753_G_0_Rennes (GenBank accession JX425879), collected in Rennes, Northwest France, which had a mosaic structure in *pol* coincident with that of X3208, 9196_01 and X3121 (Fig 3a, Table 1) and branched with them in the ML (Fig 3b) and Bayesian (S3 Fig) trees.

For another virus, VLGC-PT-BG3 (GenBank accession AY669786), from Portugal, which grouped with CRF73_BG viruses in the V3 region (Fig 1b, S1 Fig), the full-length envelope gene is available, which was analyzed by bootscanning and with jpHMM. The analyses revealed a G/B/G recombinant structure coincident with that of X3208 and 9196_01 (Fig 3c, Table 1).

**Fig 4:**
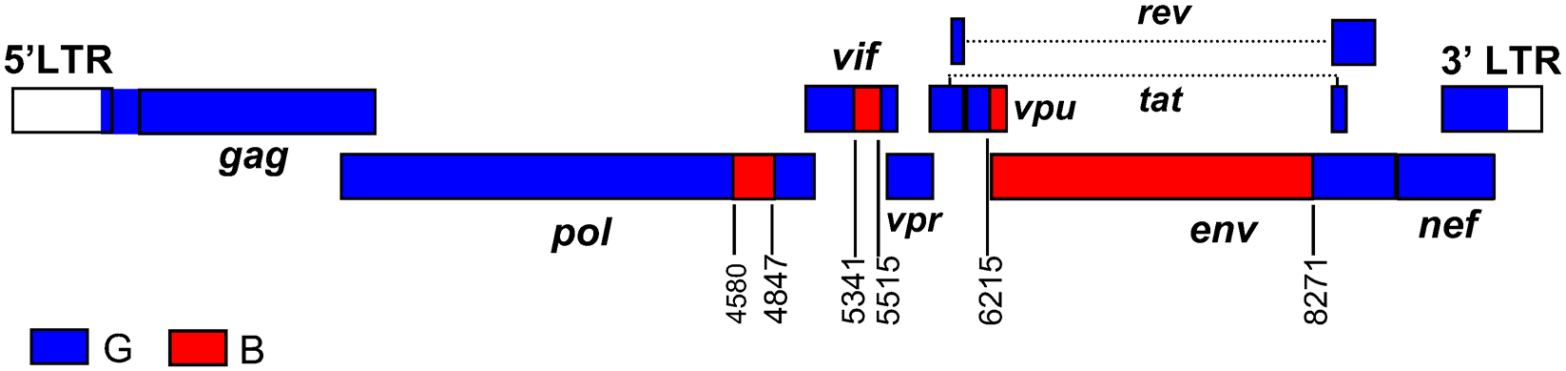
Mosaic structure of CRF73_BG. Breakpoint positions, according to HXB2 genome numeration, are indicated.

Considering the wide geographical distance between the sampling locations of the five BG recombinant viruses exhibiting identical mosaic structures (Germany, Northwest France, Portugal, and two cities in Northwest Spain 220 km. apart), it is unlikely that they are epidemiologically linked. Consequently, with the identification of five presumably epidemiologically-unlinked recombinant viruses, two of them characterized in near full-length genomes and sharing identical mosaic structures, and three analyzed in partial genome fragments, in which they cluster tightly with the near full-length genomes and show coincident breakpoints with them, the criteria for definition of an HIV-1 circulating recombinant form are met [27]. The newly identified CRF was given the designation of CRF73_BG at the HIV Sequence Database, Los Alamos National Laboratory, according to the order of discovery and the parental subtypes. Based on the analyses presented above, the inferred mosaic structure of CRF73_BG is shown in Fig 4.

### Analyses on the Relationship of CRF73_BG with CRF14_BG

**Fig 5:**
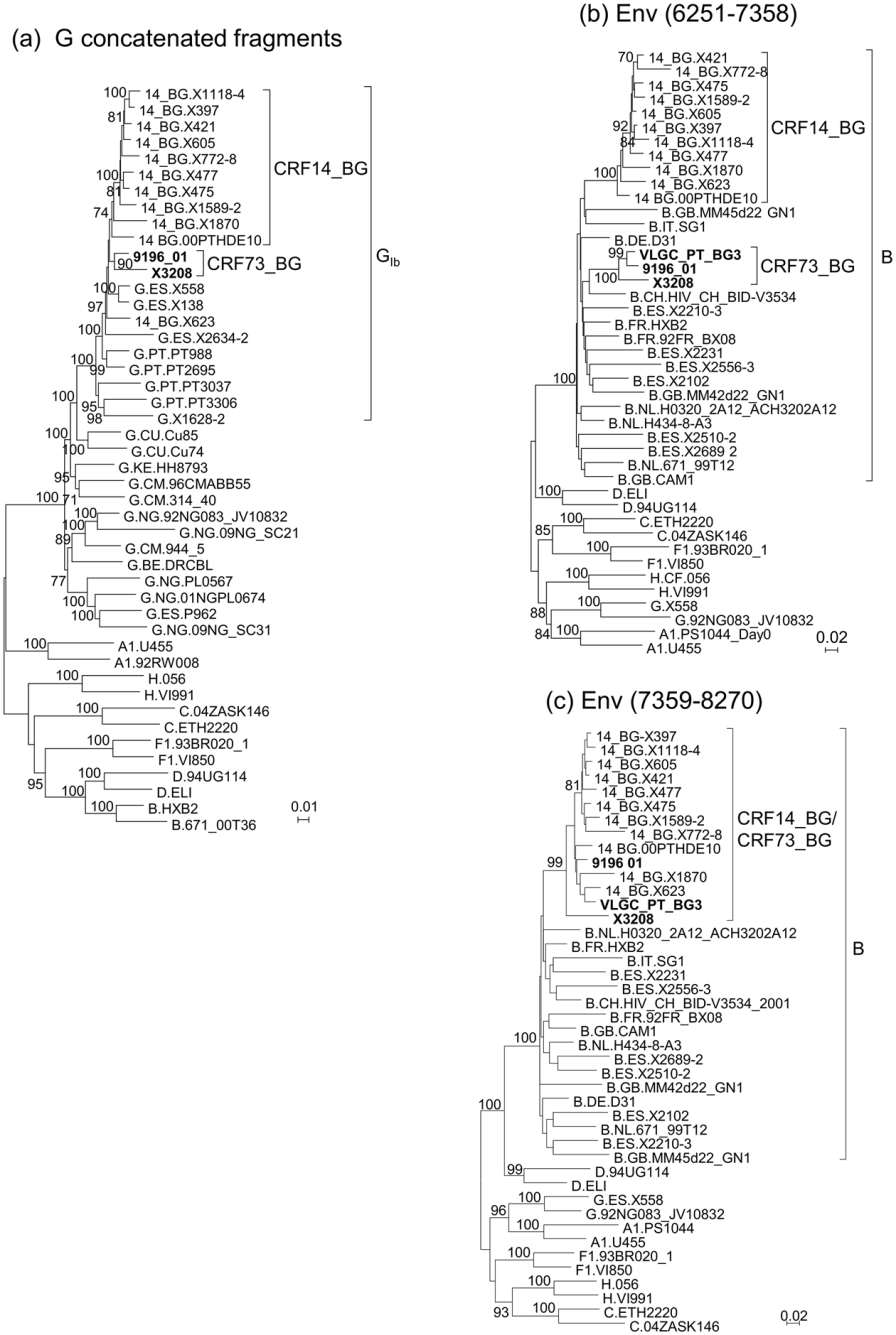
Analysis of the relationship of CRF73_BG with CRF14_BG. (a) ML tree of concatenated subtype G fragments of X3208 and 9196_01, analyzed with G_Ib_ and CRF14_BG viruses. Countries of collection of subtype G viruses are indicated with the two-letter ISO code. (b) ML tree of the 5’ *env* fragment. (c) ML tree of the subtype B 3’ *env* fragment. HXB2 positions delimiting the analyzed segments in (b) and (c) are in parentheses. CRF73_BG viruses are in bold type. Countries of collection of database viruses of subtype G viruses in (a) and of subtype B viruses in (b) and (c) are indicated with the two-letter ISO code. Only bootstrap values ≥70% are shown.

Since CRF73_BG and CRF14_BG cluster together in the full-length genome tree (Fig 2b, S2 Fig) and have one coincident breakpoint in gp41 (Table 1), we analyzed their phylogenetic relationships in different genome segments. To examine their relationship in the subtype G fragments, we constructed ML and Bayesian trees with the concatenated subtype G fragments common to both viruses, together with G_Ib_ and CRF14_BG viruses. CRF14_BG viruses, except X623, on the one hand, and both CRF73_BG viruses, on the other, formed respective clades supported by bootstrap values of 74% and 90%, respectively (Fig 5), and PP of 1 (S4 Fig), but both clades failed to cluster with each other (the node joining both clades had a bootstrap support of 29% and a PP of 0.42). We further analyzed independently three subtype G fragments, separated by the subtype B fragments in *pol* and *env* (excluding the short subtype B of *vif* in the second fragment). In each fragment, X3208 and 9196_01 failed to cluster with the clade formed by most CRF14_BG viruses (results not shown). However, it should be pointed out that X623 isolate, classified as a CRF14_BG virus, also failed to cluster with other CRF14_BG viruses in all the trees of subtype G fragments, either analyzed separately or as a concatenated sequence; we previously hypothesized that failure of subtype G fragments of X623 to cluster with CRF14_BG viruses could be due to secondary recombination with a G_Ib_ virus different from the parental of CRF14_BG [7].

In the study by Harris et al. [15], it was noticed that a portion of the subtype B *env* fragment of 9196_01, approximately the 3’ half, grouped with CRF14_BG, while the 5’ half did not. Based on this observation, and on bootscan analyses and inspection of the sequences, we constructed separate phylogenetic trees of envelope fragments spanning HXB2 positions 6251–7358 and 7359–8270, respectively. We observed, in accordance with the previous study, that X3208, 9196_01, and VLGC_PT_BG3 clustered with CRF14_BG in the 3’ fragment, but not in the 5’ fragment of *env* (Fig 5, S4 Fig). Analysis of the amino acids at the V3 loop of these viruses and of 17 other viruses from Spain and Portugal that clustered with them (Fig 1b, S1 Fig) confirmed the absence of most of the four residues reported to be characteristic of CRF14_BG viruses [10], of which none or only one were present in CRF73_BG viruses, except in one, which had two.

### Proportions of Database Viruses from Portugal Clustering with CRF73_BG

Among viruses collected in Portugal with sequences at the HIV Sequence Database [23], those clustering with CRF73_BG in the V3 region and in PR-RT (Fig 1) represent 3.2% (19 of 601) and 1.7% (12 of 709), respectively. Although clustering with CRF73_BG in partial genome segments lacking breakpoints does not ensure that the sequences are from CRF73_BG, considering that for both PR-RT and V3 fragments all three viruses for which longer sequences are available have breakpoints coinciding with CRF73_BG, it is most reasonable to assume that most, if not all, other viruses branching in the same cluster are also CRF73_BG viruses.

### Estimation of tMRCA of CRF73_BG

Using 16 PR-RT sequences for which year of sample collection was available and the Bayesian method implemented in BEAST, the tMRCA of the ancestral node of viruses of the CRF73_BG cluster was estimated in 1989.5 (95% HPD, 1982.3–1994.9).

## Discussion

The results here reported allow to identify a new HIV-1 CRF, designated CRF73_BG, whose genome mainly derives from the subtype G variant circulating in Spain and Portugal and which has three subtype B-derived fragments. The 3’ subtype B fragment of the envelope and probably its adjacent subtype G segment (considering the coincident breakpoint) are related to CRF14_BG, but the 5’ subtype B *env* fragment and most of the subtype G fragments have a different ancestry. Therefore, although its mosaic structure has some resemblance to that of CRF14_BG [7] with additional subtype B fragments, most of its genome does not appear to derive from it. Considering the greater complexity of the mosaic structure of CRF73_BG, the most parsimonious scenario would be that it derives from three parental viruses, belonging, respectively, to CRF14_BG, a G_Ib_ strain different from the parental of CRF14_BG, and subtype B. However, an alternative scenario, in which CRF73_BG would be one of the parental strains of CRF14_BG, cannot be completely ruled out.

It is important to make the distinction between CRF73_BG and CRF14_BG, since the biological features ascribed to CRF14_BG, which has the highest reported frequency of CXCR4 co-receptor usage among HIV-1 genetic forms and characteristic amino acids at the V3 loop [10], may not apply to CRF73_BG. The mosaic structure of CRF73_BG, which probably derives from at least three parental strains, including another CRF, reflects the constantly increasing genetic complexity of the HIV-1 epidemic, with CRFs giving rise to new CRFs through secondary recombination with other clades.

CRF73_BG is the third CRF reported to presumably originate in the Iberian Peninsula, after CRF14_BG [7] and CRF47_BF [28], reflecting the co-circulation of diverse HIV-1 genetic forms among the local population. CRF73_BG circulates in Portugal and, to a much lesser extent, in Spain, with sporadic cases found in Germany and France. In database sequences from Portugal, CRF73_BG represents around 2–3% viruses analyzed in V3 or PR-RT segments. The prevalence of CRF73_BG could be greater among IDUs in Lisbon, since we have noticed that 11 (39.3%) of 28 subtype B sequences of the V3 region from viruses collected among this population in the study by Esteves et al. [11] cluster with CRF73_BG (Fig 1) (these viruses correspond to ‘cluster I’, as designated by the authors). Since, in the mentioned study, subtype B viruses, as analyzed in the V3 region, were 57% of the total, and assuming that ‘cluster I’ corresponds to CRF73_BG, the estimated overall proportion of CRF73_BG viruses among IDUs in Lisbon would be 22.4%. In Spain, circulation of CRF73_BG appears to be much more limited than in Portugal. Among the seven CRF73_BG viruses collected in Spain identified by us, four were from Spanish and two from Portuguese individuals, and no data on country of origin was available for another one, and transmission route, reported for six individuals, was either through injecting drug use, in four, or sexual contact, in two (S1 Table).

The fact that CRF73_BG has been identified about a decade after the characterization of the near full-length genome of one of the viruses belonging to it and around 12 years after the description of a subtype B cluster in a partial *env* fragment formed by viruses presumably belonging to this CRF may have wider implications for the HIV-1 epidemic. First, it implies that, in addition to the currently known HIV-1 CRFs, there may be many more CRFs circulating at relatively low prevalences misidentified as URFs awaiting to be discovered, highlighting the great and underestimated diversity of HIV-1 circulating genetic forms generated through recombination, constituting a heterogeneous pool of variants with diverse biological properties that can be selected for expansion when introduced into a transmission network. And second, it underscores the importance of full-length or near full-length genome sequence analysis for a proper genetic characterization of HIV-1 strains, which, from an epidemiological and public health point of view, may be particularly relevant for the study of the numerous HIV-1 clusters which are increasingly being reported in recent years in many countries, most of which have been characterized only in partial genome segments.

## Conclusions

A new HIV-1 CRF circulating in Portugal and Spain has been characterized, derived from locally circulating strains. The newly identified CRF corresponds to a virus described ten years ago as a URF [15] and probably to a subtype B cluster identified twelve years ago in Portugal based on partial sequences [11]. This implies that, in addition to the currently known HIV-1 CRFs, there may be many more mislabeled as URFs or subtype clusters. This study highlights the need for precise genetic characterization of the HIV-1 variants circulating in a population, which allows for a better knowledge of the history and dynamics of virus propagation and of the biological features associated to these variants, which may help guide preventive efforts against the HIV-1 epidemic.

## Supporting Information

S1 Fig Bayesian phylogenetic tree of sequences of HIV-1 isolates clustering with the BG recombinant virus 9196_01 in (a) PR-RT and/or (b) the V3 region. Sequences are the same as in the ML trees of Fig 1. For this and subsequent Bayesian trees, nodes with PP = 1 are labelled with filled circles and those with PP = 0.95–0.99 are labelled with unfilled circles.
(TIF)

S2 Fig Bayesian tree of near full-length genomes of X3208 and 9196_01, analyzed with subtype G viruses from Spain, Portugal, and other countries, and CRF14_BG references. (TIF)

S3 Fig Bayesian tree of the integrase subtype B fragment of X3121 and 753_G_0_Rennes, showing clustering with 9196_01 and X3208. The sequences used for the tree are the same as those used for the ML tree of Fig 3b.
(TIF)

S4 Fig Bayesian phylogenetic trees analyzing the relationship of CRF73_BG with CRF14_BG. (a) Tree of concatenated subtype G fragments of X3208 and 9196_01, analyzed with G_Ib_ and CRF14_BG viruses. (b) Tree of the subtype B 5’ env fragment. (c) Tree of the subtype B 3’ env fragment. The sequences used for the trees are the same as those used for the ML trees of Fig 5. In (a), the node joining CRF14_BG and CRF73_BG clades is supported by a PP of 0.42.
(TIF)

S1 Table Data of HIV-1-infected individuals infected with viruses clustering with 9196_01 BG recombinant strain. (XLSX)
