# Supplementary figures and images for: Identification of an HIV-1 BG Intersubtype Recombinant Form (CRF73_BG), Partially Related to CRF14_BG, Which Is Circulating in Portugal and Spain
Source: PLoS One. 2016 Feb 22;11(2):e0148549. doi: 10.1371/journal.pone.0148549 (PMC4765764; doi:10.1371/journal.pone.0148549)

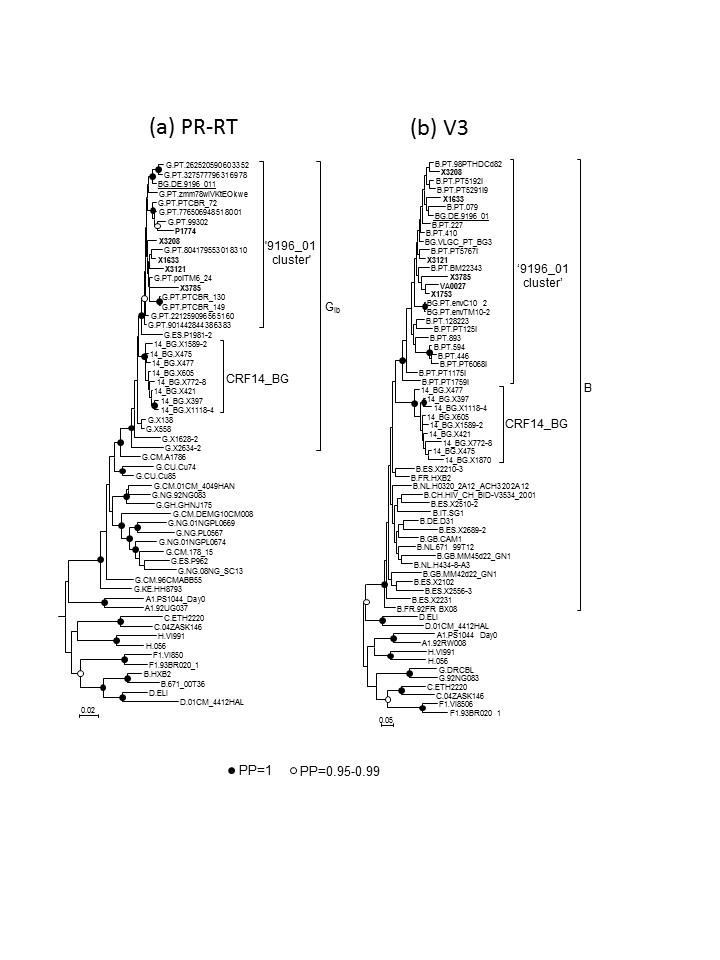

Supplement: S1 Fig — Sequences are the same as in the ML trees of Fig 1. For this and subsequent Bayesian trees, nodes with PP = 1 are labelled with filled circles and those with PP = 0.95–0.99 are labelled with unfilled circles. (TIF) [file pone.0148549.s001.tif]

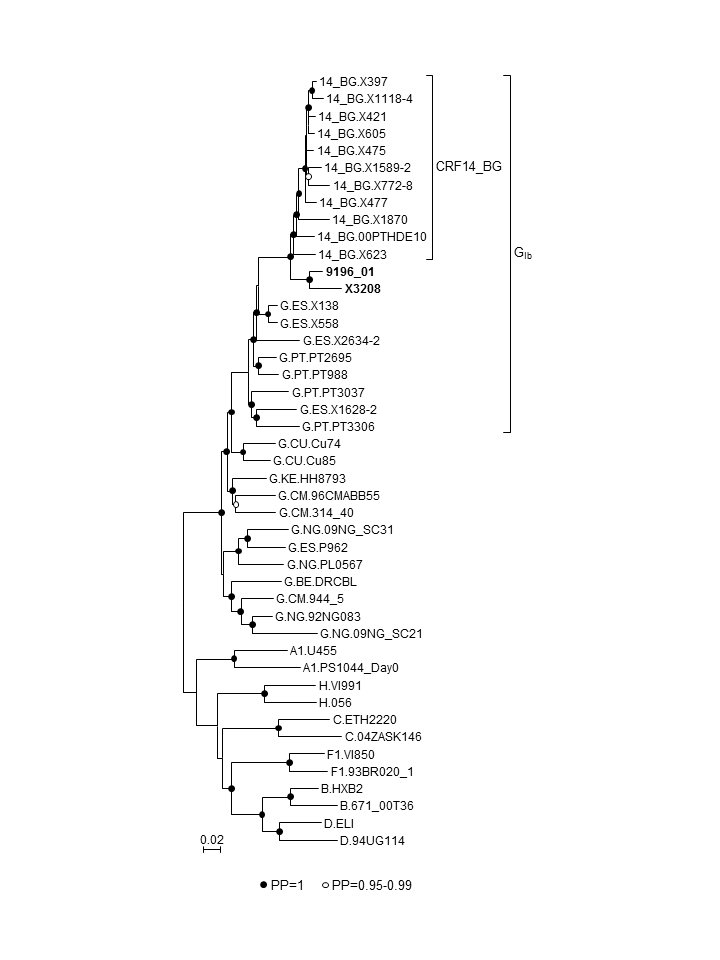

Supplement: S2 Fig — (TIF) [file pone.0148549.s002.tif]

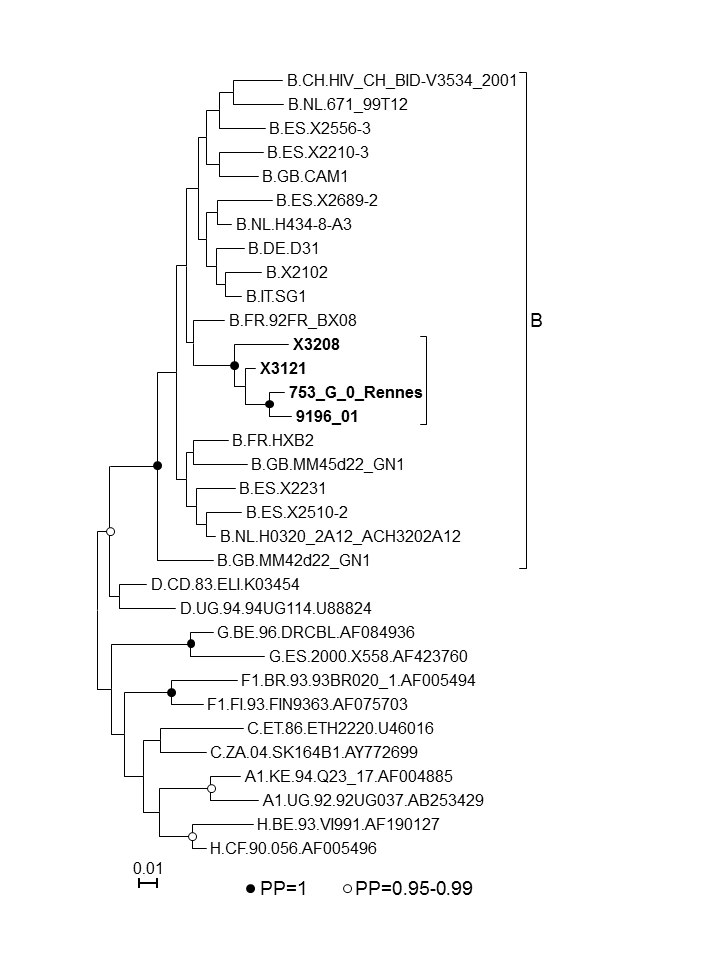

Supplement: S3 Fig — The sequences used for the tree are the same as those used for the ML tree of Fig 3b. (TIF) [file pone.0148549.s003.tif]

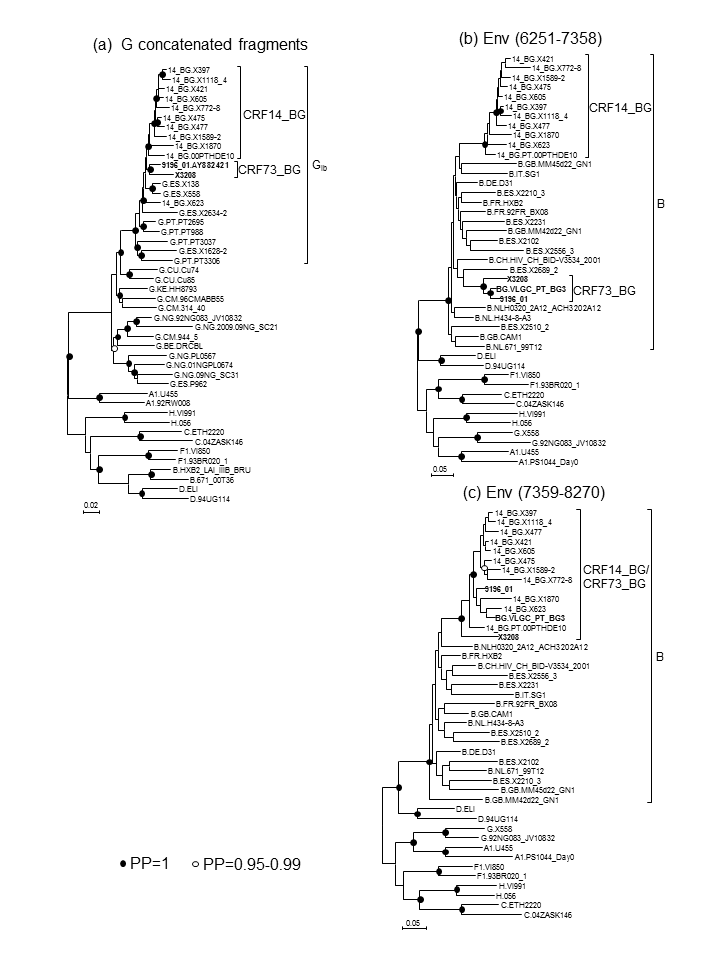

Supplement: S4 Fig — (a) Tree of concatenated subtype G fragments of X3208 and 9196_01, analyzed with GIb and CRF14_BG viruses. (b) Tree of the subtype B 5’ env fragment. (c) Tree of the subtype B 3’ env fragment. The sequences used for the trees are the same as those used for the ML trees of Fig 5. In (a), the node joining CRF14_BG and CRF73_BG clades is supported by a PP of 0.42. (TIF) [file pone.0148549.s004.tif]
